# 

**Published:** 1922-11

**Authors:** 


Supplement to February, 1924.

### THE HOSPITAL and HEALTH REVIEW

A JOURNAL FOR EVERYONE INTERESTED IN PUBLIC HEALTH, PREVENTIVE AND CURATIVE MEDICINE, NATIONAL INSURANCE AND HOSPITAL AND INSTITUTION WORK.

*Established 1886.*

LIBRARY
FEB 12 1924
SURGEON GENERAL'S OFFICE

New Series VOLUME II.

(Volume LXXII.)

---

OCTOBER, 1922, TO DECEMBER, 1923.

---

SURGEON GENERAL'S
256999
LIBRARY

LONDON:
PUBLISHED BY THE SCIENTIFIC PRESS (LIMITED), AT THE *HOSPITAL* BUILDING,
28 & 29 SOUTHAMPTON STREET, STRAND, W.C.2.

MDCCCCXXIII.

W1
HO71PL
v. 2
1922/23
c. 1

### INDEX TO VOL. II. (New Series).

(Volume LXXII).

Accidents, 7, 131
Adenoids and the White Races, 416
After-Care of Hospital Patients, 281, 307
Ainsworth-Davis, Major J. R., "The 'New Movement' in Hospital Appeals," 37
Alcohol (*see* Intoxicating Liquor)
Alexandra Day, 24
Almoners, Lady, 133, 194
America, 293, 316, 317, 351, 416, 444; Death-Rate, 3, 217, 226; Milk, 16; Leprosy, 72; Dirty Money, 162; Safety First, 195; Hospital System, 196, 351; Sleeping Sickness, 218; Sanitary Progress, 249; Health Organisations, 153, 217, 218, 286; Hospital for London, 418
Anæmia, Decrease of, 360
**APPOINTMENTS**, 405 (Oct., 1922), 24, 59, 88, 126, 152, 186, 220, 252, 288, 320, 354, 386, 414, 446
Army Medical Service, The, 331
Art of Living, The, 397
Association of Certificated Blind Masseurs, 94, 418
Australia: Hospital System, 406; "Reservoir of Disease," 415; Sydney Hospital and Auxiliary, 442

Baby Week (*see* Infant Welfare)
"Bayer 205," 134, 416
Beer, Lead Poisoning from, 378 (Oct., 1922)
Belgium: Labour Hospital, 8; Hygiene and Nursing, 173; Familial Treatment of Lunatics, 217; new Hospital, 306
Birth Control, 17, 433
Birth-Rate: Commission, 195; in France, 351; and Empire, 359
Black Country, Replanting the, 174, 194
Blind: National Institute, 32, 416; Bradford Royal Institution, 168; Association of Certificated Blind Masseurs, 94, 418; Gonorrhœal Ophthalmia in Denmark, 391
Blood-Pressure, 359, 423
Botulism, 2
Bradford: Royal Institution for the Blind, 168; Open-Air School, 177; Housing, 226; Public Health, 231, 232; new Infirmary, 378; Royal Infirmary, 414
British Charities' Association, 339
British Hospitals' Association, 211, 238, 272
British Medical Association, 322, 323, 324, 325
British Red Cross Society (*see* Red Cross)
Burnett, Sir N., Reports by, 1, 86, 274, 289, 291, 297, 404

Cadet Corps and the War Office, 98
Canada: Death-Rate, 217; Victorian Order of Nurses, 292
Cancer, 124, 160, 194, 234, 256, 285, 320, 341, 353, 357, 383, 385, 443, 445, 450; Popular Lecture on, 400
Catherine Gladstone Home, The, 426
Census Returns, 43
Clothing in Relation to Health, 434
Cocaine, 193, 244
Cod-Liver Oil, The Romance of, 182
Coles, Mr. R. J., "Regular Contributions in South London," 39
"Colour-Hearing," 424
Co-operation of Hospitals, 9, 41, 108, 141, 205, 364, 405
Cooper Hodgson, Miss H. S., "The Art of Living," 397
Co-ordination of local Health Services, 399

**CORRESPONDENCE.**
Artificial Legs for Civilians, 114
Asylum Reforms, 147
Blind Masseurs, 181
Bottle of Medicine Habit, The, 181, 211
Bread, 147, 181, 210
Cadet Corps as Health Centres, 147
Central Surgeries, 146
Co-operation in a Hospital Contributory Scheme, 58
Cottage Hospitals, 342
County Medical Service, Report of Committee of the Wiltshire Branch of the British Medical Association, 57
Dentists' Register, The, 94
Dirty Money, 211
Eye, Prescribing for the, 244
Gas Poisoning, 146
German Hospital, In a, 210
Health Work, 408

CORRESPONDENCE.—*Continued.*
Hospital Finance, 404 (Oct., 1922), 93, 114
Hospital Wards, Re-naming, 408
Housing and Land, 114
Humane Slaughter, 114, 269
Lavender for Hospitals, 314
Middle Classes in Hospital, The, 244
Milk, 342
Ministry of Health, 181, 408, 441
Nursing Homes, In the, 211, 244
Nursing of Insured Persons, The, 31, 57
Ophthalmia in Lying-in Hospitals, 181
Panel Work, 404 (Oct., 1922), 441
Probationer, The modern, 244
Public House Reform, 363, 408, 442
Radium, The Price of, 442
Tomatoes, The Value of, 314
Unemployment and the Dole, 94
Vaccination, 314
Voluntary Hospitals, 146, 441
Women's Holiday Fund, 270
Women's League of Service for Motherhood, 210

Cripples' Aid Society, North Staffs, 368

Croydon: the Whitgift Hospital, 193; Hospital Scheme, 418

Cummins, Miss A. E., "After-Care of Hospital Patients," 281

Cutaneous Injection in Germany, 225

Dangerous Drugs Act, The, 159, 196

Deacon, Mr. W., "How a Country Hospital was saved," 304

Death-Rate, The, 127, 160, 217, 352; America, 3, 217, 226; Canada, 217

Denison House Committee on Public Assistance, 98

Denmark: Sobering Result of Taxation, 390; Gonorrhœal Ophthalmia, 391; Finsen Treatment, 415

Dentistry: 156, 249, 383, 415, 417, 421, 427; Folkestone Clinic, 19; Dentists' Act, 70, 79, 94; Care of the Teeth, 132; Leeds, 137; Dental Caries, 326; Dover Clinic, 342

Diabetes (*see* Insulin)

Diphtheria: Antitoxin, 381 (Oct., 1922); Carriers, 196, 358; Schick Test in Edinburgh, 444

Dirty Money, 162

District Nursing Statistics, 19

Doncaster Regional Planning Scheme, 97, 110

Drug Habit, The, 128, 415

Drunken Driv[illegible]rs, 359

Eason, Dr. H. L., "Problems of Hospital Administration," 276

**ECHOES OF THE MONTH,** 123, 153, 183, 217, 249, 286, 315, 351, 383, 415, 443

**EDUCATION AND TRAINING:** Physical Education of Girls, 397 (Oct., 1922); Norway, 4; Health of the School Child, 17, 421, 423, 427; Physical Training, 69; Medical Inspection of Teachers, 100; Secondary Schools, 113, 315; Physically Defective Children, 145; an Open-Air School, 177; Kenya Colony, 316; the Medical Curriculum, 345; Child Psychology, 443

Eggs, Liquid, 7

Encephalitis lethargica, 5, 134, 153, 218, 384, 416

Epidemic in a Hospital, 265

Essex, Public Health in, 429

Euthanasia, 7

Ex-Service Men, 282; in Asylums, 3; Training Colony, 152

Eyes: Oculist or Optician, 256; Microscoping the living Eye, 424

Factory Inspection and Welfare Work, 380 (Oct., 1922)

Faded Records, How to restore, 424

**FINANCE AND PROGRESS, HOSPITAL** (*see also* Voluntary Hospitals), 401 (Oct., 1922), 1, 20, 22, 35, 39, 49, 50, 52, 112, 137, 141, 171, 172, 201, 202, 204, 233, 253, 266, 274, 278, 281, 289, 301, 307, 339, 357, 377, 386, 393, 401, 404, 405, 428, 431, 436, 445

Fires in Hospitals, 230, 257, 401, 405, 417

First-Aid and the Cinema Actor, 330

Flies, 68, 324

Fog, 67, 130

Food, 162, 389, 445; Poisoning, 375 (Oct., 1922), 2, 153, 323, 374; and Dirt, 154, 341, 358; Bread, 316, 352

France: Armistice Monument, 32; Hygiene in Schools, 316; Children's Mountain Home, 316; Declining Birth-Rate, 351; Rejuvenation, 415

Fumigation of Ships, 380 (Oct., 1922), 7

Gas: Dangers, 97; Values, 154; Poison Gases in Disease, 249

General Charities' Needs and Appeals, 450

Germany: Doctors' Fees, 379 (Oct., 1922); Health, 99; Dr. Ponndorf's Claims for Cutaneous Injection, 225; Conditions in, 286; Censorship of Medicines, 292

**GIFTS AND BEQUESTS,** 401 (Oct., 1922), 23, 87, 92, 234, 318, 332, 382, 384, 400

Goitre and Iodine, 415

Gonorrhœal Ophthalmia in Denmark, 391

**GOVERNMENT PUBLICATIONS,** 378, 380, 402 (Oct., 1922), 24, 59, 88, 122, 127, 154, 160, 224, 373, 374, 419, 427

Greece, Terrible Condition of Refugees in, 154

Green, Mr. H. G., "The Years that Count," 262

Hair Dyes, The Dangers of, 6

Hambleden, Viscount, "Hospital Finance in its Relation to Approved Societies," 278

Hamilton, Mr. G., "Evolution of the Voluntary Hospital," 207

Harris, Mr. R. W.: "Medicines and Meddlesomeness," 391 (Oct., 1922), 12; "Panelphobia," 44; "Organisation of Panel Practice," 104; "A Fair Chance for the Panel System," 139; "Insurance Scraps of Paper," 164; "Panel Pay and Extended Services," 200, 229, 261; "Pity the poor Panel Patient," 303; "September and the Panel Doctor," 333; "If the Doctors Refused," 365

Head-to-Foot Bed Arrangement, 327

Health (*see also* Public Health), 203, 241, 383, 397, 432, 446; Weeks, 406 (Oct., 1922), 18, 154, 321, 400, 446; Lectures in Prisons, 18, 385; and Noise, 294; Co-ordination of Services, 399

Heliotherapy, 326, 328, 422, 443
Hill, Prof. L., "Clothing in Relation to Health," 434
Homes for Aged Nurses, The Samuel Welsh, 409
Hospital Finance (*see* Finance)
Hospital Management, 82, 276
Hospital Needs and Appeals, 60, 447
Hospital News (*see* News)
Hospital Nursing and Midwifery Exhibition, 145, 163, 187, 206
Hospital Officers' Association, 137
Hospital Saturday Committee, The, 400 (Oct., 1922)
Hospital Saturday Fund, 194, 199, 266, 418
Hospital Sunday Fund, 353

**HOSPITALS.**

METROPOLITAN :

Charing Cross Hospital, 318, 447
City of London Maternity Hospital, 119, 319, 409, 448
Greenwich, Miller General Hospital, 414, 449
Guy's Hospital, 296, 319, 385, 447
King's College Hospital, 297, 377, 447
London Hospital, 40, 60, 92, 107, 171, 385, 426, 445, 447
Maudsley Mental Hospital, 401 (Oct., 1922), 141, 142
Middlesex Hospital, 52, 53, 71, 144, 308, 317, 394, 402
National Hospital for Diseases of the Heart, 319
Poplar Hospital for Accidents, 385
Queen Charlotte's Hospital, 319, 449
Queen Mary's Hospital, West Ham, 340, 449
Royal Northern Hospital, 133, 205, 314, 318, 339, 382, 431, 448
Royal Waterloo Hospital for Women and Children, 62, 92
St. Bartholomew's Hospital, 227, 259
St. Luke's Hospital, 71
St. Mary's Hospital, 319, 448
St. Thomas's Hospital, 262
University College Hospital, 251, 267, 448
Westminster Hospital, 307, 364, 448

PROVINCIAL :

Alton Cripples' Hospital, 445
Bangor : Carnarvon and Anglesey Infirmary, 173, 266, 425 ; St. David's Hospital, 300
Birmingham, Highbury Hospital, 257, 401, 417
Bradford Royal Infirmary, 414
Bridgwater Hospital, 304
Brighton, Royal Sussex County Hospital, 401, 403 (Oct., 1922), 112, 169, 171, 204, 308, 398, 445
Cirencester Hospital, 202
East Suffolk and Ipswich Hospital, 332

HOSPITALS.—*Continued.*
Gloucester Royal Infirmary, 386
Hastings, East Sussex Hospital, 113, 240, 437
Ipswich (*see* East Suffolk)
Keighley, Victorial Hospital, 418
Leicester Royal Infirmary, 141
Lincoln County Hospital, 51
Liverpool Royal Infirmary, 386
Manchester : Babies' Hospital, 337 ; Royal Infirmary, 234, 319
Newcastle, Royal Victoria Infirmary, 405 (Oct., 1922), 319, 393
Nottingham General Hospital, 307, 414
Sheffield Royal Hospital, 234
Shrewsbury Hospital, 9
Stourbridge, Corbett Hospital, 401 (Oct., 1922)
Sunderland Royal Infirmary, 319, 417
Wrexham War Memorial Hospital, 307

SCOTLAND :

Edinburgh, Royal Infirmary, 401 (Oct., 1922), 113
Glasgow : Royal Hospital for Children, 386 ; Royal Infirmary, 112, 386, 405 ; Royal Maternity Hospital, 318
Insch and District Hospital, 8

COLONIAL AND FOREIGN :

British Seamen's Hospitals, 72, 233
France, Châlons Maternity Hospitals, 218
Singapore, St. Andrew's Mission Hospital, 375
Sydney, Royal Alfred Hospital, 442

Hospitals, Combined Appeal for London, 22, 85, 100, 112, 250
Hospitals, Future of the, 21, 33, 107
Hospital Saving Association Schemes (*see also* Finance), 35, 37, 39, 97, 137, 141, 172
Housing Problem, The, 380, 387 (Oct., 1922), 10, 67, 74, 95, 124, 125, 129, 135, 152, 157, 184, 189, 203, 220, 226, 252, 288, 324, 336, 354, 357, 366, 420, 429
Humour and Tragedy of Hospital Life, 46
Hygiene : in French Schools, 316 ; Institute of, 318 ; London School, 334, 445 ; Education in, 444

Illegitimate Child, The, 132, 160
India : Nursing in, 26 ; Ambulance Work in Bombay, 173 ; Baby Week, 417

**INFANT WELFARE**, 386, 406 (Oct., 1922), 7, 94, 106, 202, 231, 232, 258, 262, 293, 316, 351, 361, 373, 383, 416, 422, 427, 430 ; National Baby Week Council, 422 ; New Zealand, 391 ; Transvaal, 391 ; York, 406 ; India, 417
Infectious Diseases, 125, 374 ; Hospital Management and Treatment of, 398 (Oct., 1922) ; Epidemics and Holiday Centres, 5 ; Suggested Bed Arrangement, 327
Influenza, 138
Inman, Mr. P., "Humour and Tragedy of Hospital Life," 46
Insanity (*see also* Lunacy) : Incipient, 221 ; Puerperal, 242 ; Maudsley Hospital, 401 (Oct., 1922), 141, 142,
Insulin, 78, 125, 130, 250, 257, 271, 276, 317, 374, 418, 435, 445
**INSURANCE, NATIONAL HEALTH**, 391 (Oct., 1922), 13, 44, 45, 80, 81, 104, 109, 125, 139, 140, 152, 164, 170, 184, 197, 200, 203, 212, 220, 223, 229, 230, 237, 252, 261, 271, 288, 294, 303, 313, 320, 333, 338, 365, 369, 373 ; Consolidation Act, 324 ; Ultimatum to Panel Doctors, 395, 432 ; Panel Court of Enquiry, 446 ; Approved Societies, 141, 171, 233, 278, 313, 338, 369, 396, 432
Intelligence : Measuring, 392 ; and Brain, 444
International Health Board, The, 334, 421 ; Annual Report, 433
Intoxicating Liquor : Lady Astor's Bill, 159, 193, 292 ; and Cider, 193 ; and Liver Disease, 225 ; and Taxation in Denmark, 390
Ireland : Public Health, 166, 353, 403 ; Hospital Sweepstakes, 340
Isolation Hospitals, The Question of, 436

James, Colonel S. P., "Mosquitoes and Malaria in England," 19
Jenner, Dr. E., 6, 97, 198

Kennedy, J., "The Lady Almoner," 133
King Edward's Hospital Fund, 84, 112, 256, 404, 428 437

Labour Party: and Health, 111; and Housing, 420
*Lancet* Centenary, 390
Lead Poisoning in Beer, 378 (Oct., 1922)
League of Mercy, The, 314, 352
League of Nations, The, 159, 351
League of Remembrance, The, 302, 435
Leeds: Hospital Schemes, 378, 437; Health Exhibition, 432
Legal Assistance for the Poor, 392
Leicester, Public Health in, 335, 361
Leprosy: U.S. America, 72; New Treatment for, 249; British Empire Leprosy Relief Association, 293; India, 315
Life Assurance: and Pensions, 99; and Surgery, 391
Light and Disease, 153, 183
Liverpool: School of Hygiene, 70; Wonderful Health Statistics, 389
Livingstone College, 89, 287
Longevity: new Elixir of Life, 4; and Pensions, 99; and Montaigne, 183; Rejuvenation, 415, 444
London Medical Exhibition, 418
**LUNACY**, 125
- Asylums: Ex-Service Men in, 3; Reforms in, 156
- Belgian Colony, 217
- Dorset Mental Clinic, 437
- Mental After-Care Association, 177, 450
- Mental Hospitals' Co-operation, 217
- Mental Treatment Bill, 221, 235
- Mentally Deficient: Besford Court Catholic Home, 407; Boarding-out Scheme, 415
- Mental Hospitals: Maudsley, 142; Leicester, 318; Bethlem, 326; Lancaster, 436

Malaria in England, 19, 337
Maternity and Child Welfare, 73, 168
Measles, The Germ of, 364
Medical Curriculum, The, 345
Medical Missions, 242
Medical Officers of Health, 132, 318, 322, 324, 329
Medical Schools, A List of, 346
Medical Society of London, The, 217, 230
Medical Women's International Association, 42
Medicines and Meddlesomeness, 391 (Oct., 1922), 12
Mental Deficiency, etc. (*see* Lunacy)
Metropolitan Asylums Board, 328
Middle Classes in Hospital, The, 192, 224, 275, 280
Milk, 134, 135, 153, 162, 362, 372, 416; National Conference, 385 (Oct., 1922), 385; Regulations, 376, 386 (Oct., 1922), 15, 16, 68, 103, 125, 152, 250, 423; Micrococci in Condensed Milk, 320; Ideal Method in Hospital Supply, 370; Pasteurisation Conference, 390, 446
Ministry of Health, 11, 67, 68, 132, 159, 203, 291, 355, 378, 387, 417, 421; Annual Reports, 373, 394; Ultimatum to Panel Doctors, 395, 432; Panel Court of Enquiry, 446
Ministry of Pensions, 3; Hospital Fires, 257, 401
Morris, Mr. E. W., "Mass Contributions in London," 35
Mosquitoes: and Malaria in England, 19; destroyed by Minnows, 315
Music and Healing, 43

Neurosis, 393 (Oct., 1922), 225, 243
Newman, Sir G., Report by, 421, 423, 427
**NEWS, HOSPITAL AND HEALTH**, 23, 25, 53, 86, 121, 155, 185, 219, 250, 287, 317, 352, 384, 417, 445
Nobel Prizes for Medicine, 1922-1923, 445
Norwich, Public Health in, 263
**NOTES AND COMMENTS**, 97, 129, 159, 193, 223, 255, 291, 323, 357, 389, 421
Nottingham, Public Health in, 9
Nursery Schools, Conference on, 202, 251
Nursing Profession: Scarcity of Probationers, 406; Samuel Welsh Homes, 409; Co-operation with Provident Scheme in Sussex, 436

**OBITUARY**: Boyd Carpenter, Mr. H. J., 287; Christian, H.R.H. Princess, 255; Holder, Sir J., 250; Murphy, Sir S., 224; Nordau, M., 129; Pawling, Miss, 352; Röntgen, W., 129; Teale, Mr. T. P., 422; Treloar, Sir W., 358, 417
Occupational Therapy, 223, 324
Old-Age Pensions, The Barnet Case, 377 (Oct., 1922)
Open-Air: Hospital Ward, 27 School, 177
Our Hospitals—To-day and To-morrow, 33
"Our Ostriches," 433
Outlawry of Disease, 433

**PARLIAMENTARY QUESTIONS AND ANSWERS**, 89, 125, 152, 184, 220, 252, 288, 320, 354
Patients, Paying (*see also* Finance), 202, 253, 280, 294, 339, 377, 406
Penal and Prison Systems, Our, 394 (Oct., 1922)
Pensions in Hospitals, 177
People's League of Health, The, 18, 67, 352, 385, 446
Plastic Surgery, 443
Points from Hospital Reports, 319, 386, 414
Poisons: Food, 375 (Oct., 1922), 218; and Colours, 381 (Oct., 1922), 218, 286; from Beer, 378 (Oct., 1922); from Glass, 217; from Furs, 226
Poor-Law, 8, 53, 171, 291, 328, 373; Officers' Association, 19; Public Health Service, 71; Paying Patients, 377, 406
Portsmouth, Public Health in, 73
Presentations to Hospital Organisers, 182
Prince of Wales, H.R.H. The, 266, 296, 307, 314, 425, 445
Prison System, Our, 394 (Oct., 1922)
**PUBLIC HEALTH, THE**, 388 (Oct., 1922), 65, 67, 123, 125, 126, 130, 131, 132, 152, 184, 220, 320, 324, 341, 354, 373, 399, 432; Czecho-Slovakia, 100; Visit of Foreign Officers, 159; Ireland, 166; Scotland, 374; Scarborough Congress, 241; Annual Report, 394. Interviews with Authorities: Sheffield, 386 (Oct., 1922); Nottingham, 9; Portsmouth, 73; West Riding, 105, 135; Middlesex, 167; Bradford, 231; Norwich, 263; Yarmouth, Great, 299; Leicester, 335, 361; Essex, 429
Public Relief, Co-ordination of, 98, 129

Radium (*see* X-Rays)
Rats, 257, 389, 443
Red Cross: British, 11, 27, 445, 450; Report, 363; Norwegian Hospital Ship, 315
Regional Planning Schemes: Doncaster, 97, 110; Deeside, 295
Registration of Nurses, State, 109, 251, 317, 385
Rejuvenation, 415, 444
"Relay" Automatic Telephone Service, 32, 156, 298
Reports Received, Official, 405 (Oct., 1922), 24
Research Work, 98

REVIEWS OF BOOKS:
Abdominal Surgery, Manual for Nurses on, 311
Adenopathie Traches - Bronchique Simple chez l'Enfant, 120
Adventures of a Private Nurse, 285
Adventures of a South African Nursing Sister, 30
American College of Surgeons, 1922, etc., 216
Anæmia: Its Causes and Modern Treatment, 248
Anæsthetics, Handbook of, 440
Anatomy and Psychology for Junior Nurses, 29
Anatomy and Psychology, Textbook of, 285
Animal Parasites and Human Disease, 381
Arab Medicine and Surgery, 148
Art of Feeding the Invalid and Convalescent, 440
Asthma, 412
Bacteriology, Textbook of, 29
Braganza Necklace, The, 119
British Nurse in Peace and War, The, 343
Burdett's Hospitals and Charities, 151
Cancer: Fallacy, Theory and Fact, 379
Cancer, Le Problème du, 90
Causes of Heart Failure, 248
Central Midwives' Board, Examination Questions and Model Answers, 92
Cercles Vicieux en Pathologie, Les, 413
Chemical Lecture Diagrams, Modern, 151
Chemistry for Beginners and Schools, 120
Chloroform Anæsthesia, 214
City of London Maternity Hospital: A Short History of, 119
Common Sense in the Nursery, 151
Conditions of Nervous Anxiety and their Treatment, 213

REVIEWS OF BOOKS.—*Continued.*
Conception Control, 120
Conflict and Dreams, 284
Contraception: Its Theory, History and Practice, 379
Contribution to our Knowledge of the Clinical Course and Duration of Fatal Lung Tuberculosis, 180
Coué: My Method: including American Impressions, 381
De Arte Phisicale et de Cirurgia, 148
Dental Radiology, 30
Diabetes mellitus: Clinical Treatise on, 91
Diet for Men, 310
Diet of Women, 178
Diseases of the Ear, Practical Handbook of, 413
Dispensing Made Easy, 119
Directory of Practising Midwives and Maternity Nurses, 413
Dosage Tables for Deep Therapy, 54
Draped Idols, 285
Early Diagnosis of the Acute Abdomen, 382
Electrical Action of the Human Heart, 56
Electric Ionisation, 120
Elephant Man and other Reminiscences, The, 178
English Physicians of the Past, 413
Experiences of an Asylum Patient, 215
First-Aid X-Ray Atlas of Fractures and Dislocations, 151
Folklore in the Old Testament, 410
Functional Nervous Diseases: Their Classification and Treatment, 56
Getting ready to be a Mother, 284
Glycosuria and Diabetes, Modern Methods in the Diagnosis and Treatment of, 120
Golden Rules of Dental Mechanics, 440
Gold-headed Cane, The, 118
Great and Small Things, 214
Greek Biology and Greek Medicine, 148
Guy's Hospital Reports, 311, 440
Gynæcological Nursing, Notes on, 29
Health and the Human Spirit, 411
Hints to Probationer Nurses in Mental Hospitals, 180
History of Medicine in its Salient Features, 310
History of the Great War, 245
Hodgkin's Disease, 413
House We Ought to Live In, The, 343
How to Become a Nurse, 311
How to Start and Equip a Nursing Home, 248
Hygiene and the Public Health, 382
Hygiene of Marriage, 379

REVIEWS OF BOOKS.—*Continued.*
Hypnotism and Treatment by Suggestion, 381
If Britain is to Live, 247
Index of Prognosis and End Results of Treatment, 91
Influenza, 30
Intensive Treatment of Syphilis and Locomotor Ataxia by Aachen Methods, 54
International Union against Tuberculosis: Transactions of the Second International Conference, 55
Intestinal Stasis, A Study of, 54
Kettle Songs, 56
Laboratory Manual of Biological Chemistry, 90
Life and Work of Sir James Kay-Shuttleworth, 179
Liverpool Social Workers' Handbook, 248
London Doctors and Dental Surgeons, 440
Looking On, 54
Magic, Anthropology, and Religion, 117
Manchester: History of St. Mary's Hospitals, 1790-1922, 381
Marionettes and How to Make Them, 440
Massage in Fractures and Injuries to Joints, 381
Medical and Dental "Who's Who," 180
Medical and Nursing Homes Directory, 248
Medical Aspects of Old Age, Some, 150
Medical Climatology of England and Wales, 439
Medical History of St. Marylebone, 216
Medical Insurance Practice, 28
Medical Practice in Africa and the East, 311
Medical Practice in Otago and Southland in the Early Days, 91
Memoirs: with a Full Account of the Great Malaria Problem and its Solution, 309
Midwifery, Textbook of, 149
Mistakes and Accidents of Surgery, 117
Moorland Patients, My, 91
National Health Policy, A, 311
Nauheim Treatment of Diseases of the Heart and Vessels in England, 283
Nursing and Nursing Education in the United States, 343
Nursing Mirror Pocket Encyclopædia and Diary, 120
Nursing of Diseases of the Eye, 285

REVIEWS OF BOOKS.—*Continued.*
Nursing of Infectious Diseases, Lectures upon the, 285
Nursing of Surgical Tuberculosis in Children, 413
Nursing, Pocket Cyclopædia of, 382
Nutrition of Mother and Child, 311
Observations and Reflections on Wild Creatures, 310
Obstetrical Nursing, 149
Obstetrics for Nurses, 149
On the Edge of the Primeval Forest: Experiences and Observations of a Doctor in Equatorial Africa, 64
Oto-Rhino-Laryngology, 120
Our Unconscious Mind and How to Use It, 247
Persian Sketches, 118
Pharmacology and Therapeutics, Textbook of, 410
Physiology, Handbook of, 412
Physiology in surgical and general Practice, The new, 151
Plague, Pestilence and Famine, 411
Pneumonia, 30
Poetic Mind, The, 28
Poisonous Plants of all Countries, 216
Post-Mortem: Essays historical and medical, 283
Practical morbid Histology, 382
Practical Physics, 150
Preventive Medicine and Hygiene, 90
Psychology and mental Hygiene for Nurses, 92
Psychology of the Criminal, 119
Psycho-Therapy, A Manual of, 438
Radiology for Nurses, Notes on, 440
Rainbow, The: A Drama of Light, 151
Register of Nurses kept by the General Nursing Council of Scotland, 440
Relationship of Health to the Psychical and Physical Character in School Children, 411
Rheumatism and Gout, 120
Royal Devon and Exeter Hospital, The, 55
Royal Victoria Infirmary, Newcastle-on-Tyne, Pharmacopoeia of the, 151
Safe Marriage, 54
St. John of Jerusalem, A short History of the Order of, 119
Salisbury Infirmary, History of, 382
School of Salerno, The, 439
Scottish Voluntary Hospitals, The, 215
Settlements and their Outlook, 151
Sex Problems and their Solution, 379
Silver Lining, A, 382
Skin Diseases, 216
Social Life and the Crowd, 246

REVIEWS OF BOOKS.—*Continued.*
Stewart Cunningham, Sir Henry, 216
Students' Pocket Prescriber, The, 56
Suggestion and Common Sense, 120
Suggestion and mental Analysis, 55
Surgical Tables; Obstetric Tables, 180
Synopsis of Medicine, A, 151
Talks on Psycho-Therapy, 381
Thèrapeutique du Nourrisson en Clientèle, 382
Treatment of Injuries of the Peripheral Spinal Nerves, 30
Tuberculosis, A simple Treatment for, 380
Tuberculous Worker, The, 344
Urgent Surgery, 284
Vaccination, The Student's Guide to, 248
Ventilation of Public Buildings, 413
Vigil of Hope, The, 413
Visual Illusions: Their Causes, Characteristics and Applications, 309
Vital Factors of Food, 246
Vitamins: A critical Survey of the Theory of accessory Food Factors, 438
Rockefeller Foundation, The, 218, 226, 251, 267, 334, 421, 433, 445
Royal Sanitary Institute, 406 (Oct., 1922), 100, 287, 385, 445; Congress, 341
Royal Society, The, 241
Russia, Conditions in, 6, 94, 217, 383

Sanitary Inspectors' Association, 366; Conference, 382 (Oct., 1922)
Sanitation, The Horrors of, 306
Scientific Novelties Exhibition, 437
Scotland, 385; Aberdeen Hospitals Scheme, 308; Board of Health, 353, 374; Schick Test in Edinburgh, 440; Fate of the Lister Ward, Glasgow Royal Infirmary, 112, 405
Sheffield, 255, 317; Public Health, 386 (Oct., 1922); Hospitals (Contribution) Scheme, 52, 417, 436; Sewage Works, 76; Health Lectures, 141, 390, 434; British Hospitals' Association Conference, 211, 238, 255, 272, 281, 401; Joint Hospitals' Council Report, 377
"Shell-Shock," 393 (Oct., 1922)
Slaughter, Humane, 389
Sleeping Sickness (*see* Encephalitis lethargica)
Smallpox, 43, 70, 97, 123, 290, 323, 351
Smoke Nuisance, The, 385 (Oct., 1922), 67, 222, 249, 325, 341, 366
Social Evenings in Hospital, 144
Spiritual Healing, 356
State Children's Association, 19
Sterilisation of Undesirables, 17, 124
Stone, Mr. J. E., "Control of Hospital Expenditure," 20; "Hospital Accounts and Finance," 50, 201, 301
Street Collections in London, 233
Sunday Games, 381 (Oct., 1922)
Swindon, Urgent Hospital Needs, 376, 377, 445

Taxes and Hospitals, 266, 292
Teeth (*see* Dentistry)
Television, 422
Thirst, 392
Thyroid Gland, The omnipotent, 360
Tomato, The Value of the, 256
Tonsils, Effects of removing, 161
Town Planning: Acts (1909, 1919), 75; Doncaster, 97, 110; Deeside, 295
Training (*see* Education)
Treloar, Sir W., 129; Death of, 358
**TUBERCULOSIS**, 386 (Oct., 1922), 3, 55, 74, 124, 129, 167, 197, 220, 230, 288, 294, 325, 328, 336, 341, 353, 430; Sanatorium Industries, 379 (Oct., 1922); Sterilisation of Sputum, 70; Spahlinger's Method, 11, 330; Dreyer's Method, 255, 332, 360; Domestic Animals, 42; Medical Inspection of Teachers, 100; Some Statistics, 131; West Riding, 135; Preston Hall Training Colony, 152; Industrial Relationship, 161; Results of Sanatorium Treatment, 195; French Methods, 293, 316
SANATORIA:
Cefyn Mably, Glam., 100
Grindon Hall, Sunderland, 318
Heatherwood Hospital, Ascot, 282
Hertford Hill, Warwick, 417
Kelling, Norfolk, 379 (Oct., 1922)

Unemployment, 67, 203, 205 and Cleanliness, 255

Vaccination, 6, 125, 183, 212, 336, 357, 409
Venereal Disease, 74, 136, 153, 258, 268, 446
Visiting Nurses' Directory, The, 92
Vitamins, 161, 256
Voluntary Hospitals (*see also* Finance *and* News), 152, 158, 198, 223, 233, 234, 252, 280, 289, 297, 357, 374, 404, 428; Annual Reports, 86, 141; Evolution, 207; Australia, 406
Voluntary Hospitals' Commission, 122, 165, 205, 233, 367

Waring, Mr. H. J., "Future of Voluntary Hospitals," 280
Welwyn Garden City, Ltd., 258
Whist and Charity, 401 (Oct., 1922), 156
Winter Distress League, The, 205

X-Rays and Radium, 129, 153, 154, 234, 292, 385, 391, 417, 445

Yarmouth, Great, Public Health in, 299
Yorkshire: Public Health in West Riding, 105; Proposed Orthopaedic Hospital, 406

Printed by W. SPEAIGHT & SONS, LTD., 98 & 99 Fetter Lane, E.C. 4

### RECENT APPOINTMENTS.

### Public Health.

Dr. J. V. A. Simpson, M.D., B.S., M.R.C.S., L.R.C.P., D.P.H., to be Assistant School Medical Officer to the City of Leicester Education Committee.

Dr. Marion Knowles, M.B., Ch.B. (Leeds), late house physician, children's department, at Leeds General Infirmary, to be Medical Officer for Maternity and Child Welfare to the County Borough of Derby.

Miss Kathleen L. Cass, M.B., Ch.B., M.R.C.S., L.R.C.P., late Assistant Medical Officer of Health for Maternity and Child Welfare to Lindsey C.C., to be Assistant Medical Officer of Health and School Medical Officer to the City and County Borough of York.

### Institutional.

Dr. Bessie Goodson, of Manchester, to be woman medical officer to Swansea Corporation.

Dr. Robert Matthew Brontë, to succeed Dr. Bernard Spilsbury, as pathologist to the Home Office.

Dr. George Norton Bartlett, B.S., M.B., M.R.C.S., L.R.C.P., late Medical Superintendent of Exeter City Mental Hospital, to be Medical Superintendent of Mickleover County Asylum (Derbyshire C.C.).

### GOVERNMENT PUBLICATIONS.

[Copies can be purchased through any bookseller or direct from H.M. Stationery Office, at Imperial House, Kingsway, W.C.2; 28 Abingdon Street, S.W.1; 37 Peter Street, Manchester; 23 Forth Street, Edinburgh; and 1 St. Andrew's Crescent, Cardiff. The prices quoted include postage.]

### Command Papers.

(1749.) Scottish Board of Health. Sale of Milk in Scotland. Inter-Departmental Committee Report. 3s. 2d.

### Statutory Rules and Orders.

(1001.) Food Control Order, September 1, 1922. 1½d.

(1001x) Memorandum on the Sale of Food Order, 1921. 1½d.

(Draft.) National Health Insurance. Medical Benefit. Amendment Regulations (No. 2), 1922. (August 11). 2½d.

(1035.) Housing, England. Ministry of Health (Temporary Relaxation of Building Bye-laws) Regulations (September 16, 1922). 1½d.

(1080 S. 51.) National Health Insurance. Medical Benefit Amendment Regulations (Scotland), 1922. (No. 2). October 1, 1922, made by the Scottish Board of Health. 1½d.

### Stationery Office Publications.

Ministry of Health :—

Persons in Receipt of Poor Law Relief in England and Wales. Quarterly Statement, April-June, 1922. 5d.

Circular (342). Botulism. To Sanitary Authorities. September 19, 1922. 1½d.

Botulinus Antitoxic Serum. Instructions as to Use. 1½d.

Lunacy and Mental Deficiency. Control, Board of. Eighth Annual Report. 1921. 7s. 9d.

### OFFICIAL REPORTS RECEIVED.

**(Year to Dec. 31, 1921, unless otherwise stated) :—**

London Spectacle Mission Society.

Glamorgan County Lunatic Asylum. Bridgend.

United States Public Health Reports (August, 1922; Nos. 32-34).

South-West Lancashire and Cheshire Joint (Prescriptions) Committee.

Roxburgh District Board of Control (Lunacy). Year to May 15, 1922.

Mission to Lepers, 33 Henrietta Street, Covent Garden, W.C. 2 (6d.).

Record of the Save the Children Fund. Vol. III, No. 1 (third quarter, 1922).

Cremation Society of England, 52, New Cavendish Street, W.1. (1s., post free, to non-subscribers.)

County Borough of Birkenhead, Annual Report of the Medical Officer of Health, Dr. D. Morley Mathieson.

County Borough of Bournemouth. Annual Report of the Medical Officer of Health, Dr. A. D. Edwards.

Bibliography of Hookworm Disease (Publication No. 11). Rockefeller Foundation, International Health Board, New York City.

Third Annual Report on the Voluntary Hospitals in Great Britain (excluding London), 1921. By Sir Napier Burnett, K.B.E., M.D., F.R.C.S., F.R.C.P. (2s. 6d.).

### Hospitals and Homes.

British Dentists' Hospital.

Salop Mental Hospital, 1921. Accounts to March 31, 1922.

Louth and District Hospital and Dispensary. Year to May 31, 1922.

Nottingham General Hospital. Six months to June 30, 1922.

West Bromwich and District Hospital. Year to June 30, 1922.

Royal Albert Edward Infirmary and Dispensary, Wigan. Year to March 31, 1922.

